# Phenolic and flavonoid content of *Elaeagnus angustifolia* L. (leaf and flower)

**Published:** 2014

**Authors:** Fereshte Saboonchian, Rashid Jamei, Siavash Hosseini Sarghein

**Affiliations:** 1 *Department of Biology, Faculty of Science, Urmia University, Urmia, I. R. Iran *

**Keywords:** *Elaeagnus*, *Angustifolia*, *Flavonoid*, *Phenolic compounds*

## Abstract

**Objectives** **: **Leaves and flowers of Elaeagnus angustifolia contain phenolic and flavonoid compounds. These compounds have antioxidant properties that protect cells from oxidative damage. The aim of this study was to determine and analyze total phenolic and flavonoid content of leaves and flowers in two E. angustifolia variants using different solvents (ethanol and methanol).

**Materials and Methods**: Ethanolic and methanolic extracts of the plant leaves and flowers were prepared. Experiments were carried out to measure their phenolic and flavonoid content using two solvents. Data were analyzed using Instat-N software.

**Results**: Results showed that the amount of phenolic and flavonoid compounds in both ethanolic and methanolic extracts was higher in Fariman variant compared with Mashhad variant. Ethanolic and methanolic extracts of Fariman variant had the highest amount of phenolic compound (10.91±0.18, 10.28± 0.36 mg GAE/100gFW, respectively) and also the highest amounts of flavonoids (5.80±0.10, 3.36±0.05 mgQE/100gFW, respectively). Phenolic and flavonoids compounds were better extracted using methanol and ethanol solvent.

**Conclusions:** In both varieties and solvents, the amount of phenolic and flavonoids compounds in leaves were higher than flowers. In addition, the phenolic and flavonoids compounds were higher in Fariman compared to Mashhad variants

## Introduction

 In recent years, after proving the relationship between oxidative stress and human diseases dietary supplements and ingredients containing antioxidant properties have attracted attention (Pfannhauser et al., 2001 ▶). These compounds help to inhibit many of the oxidative reactions caused by free radicals such as superoxide, hydroxyl, and proxy nitrate and thus slow or inhibit the development of diseases (Rose et al., 1982). In other words, the production of free radicals in body cells and tissues is associated with many diseases caused during aging (Sanchez-Moreno et al., 2003). Reports indicate reverse association between dietary antioxidant-rich foods and the incidence of human diseases (Olukemi et al., 2005 ▶). 

Thus, having a diet rich in antioxidant compounds is important. The medicinal value of plants depends on their phytochemical constituents namely alkaloids, tannins, flavonoids, and phenolic compounds which are the most important compounds (Shariff, 2001 ▶). 

*E. angustifolia* (*Elaeagnus angustifolia* L.) is a European - Asian tree belonging to the family Elaeagnaceae which grows in a wide range of environmental conditions (Shafroth, 1995 ▶). It is native to northern Europe and Asia and is spread across the Himalayas. In English language, it is called Oleaster and has long elliptic leaves with sharp tips and short petioles (Zargari, 1990 ▶). Dried leaves and fruits of this species have astringent and antipyretic effects. Its flower has been used in flavoring some liqueurs. In addition, the general idea is that it also has a febrifuge effect. Iranians traditionally brewed its fruit as a painkiller for patients with rheumatoid arthritis and its flowers were traditionally used to treat tetanus. The fruits and flowers of this plant have also been applied to treat nausea, vomiting, jaundice, asthma, and abdominal distention (Zargari, 1990 ▶). Leaf extracts of *E. pungens* have been proven to be a useful herb in traditional Chinese medicine for the treatment of asthma and chronic bronchitis and are tussive and also bring up sputum (Ge, 2009 ▶). 

Antibacterial agents have been extracted from *E. glabra* bark (Nishino, 1987 ▶). Moreover, ripe fruits of *E. philippinsis* are used to treat amoebic dysentery (Perry, 1980 ▶). The most important medicinal species of this genus found in Iran is *E. angustifolia* (Elaegnus tree) (Zargari, 1990 ▶). To supply body's necessary natural antioxidants, it is recommended to use plants with high phenolic compounds (Frankel, 1999 ▶). Since reactive oxygen species are involved in the generation of most of the diseases, investigation of phenolic compounds, in order to collect and exploit them, is the subject of attention. 

Thus, the phenol and flavonoid contents of two variants of *E. angustifolia* found in Mashhad and Fariman were examined using two different solvents.

## Materials and Methods

**Plant materials**

**Figure 1:**
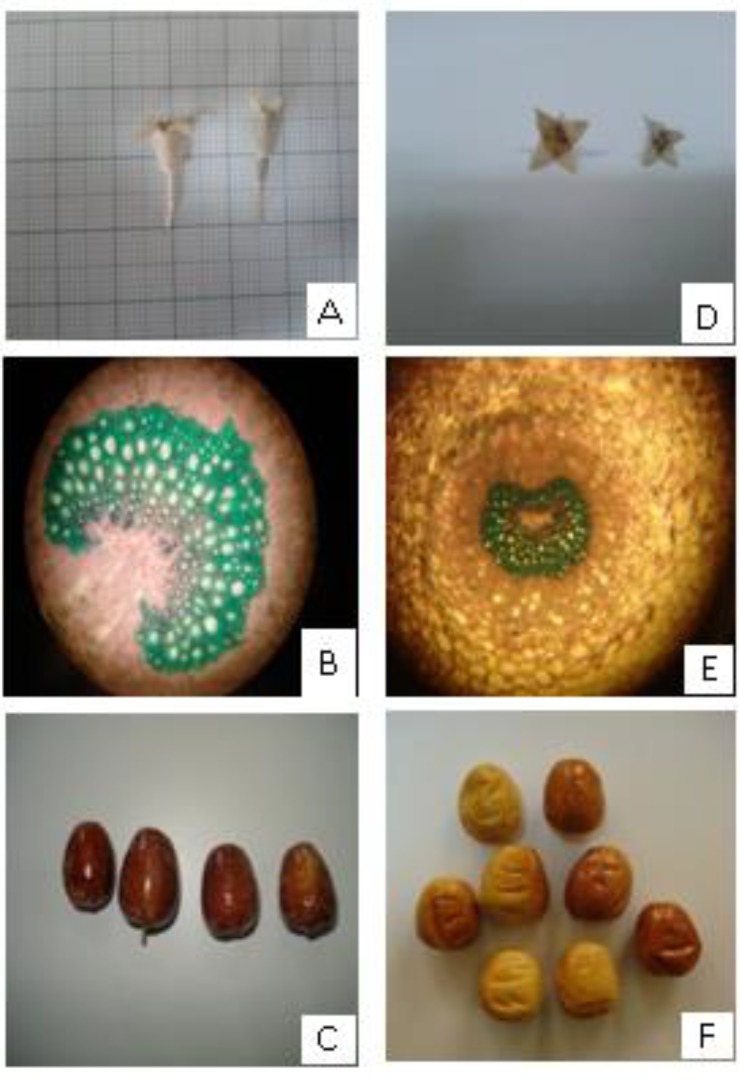
Flower, fruit and morphology of Fariman variant of *E. angustifolia* (A, B, and C) and those of Mashhad variant (D, E, and F).

The flowers and mature leaves of both variants were collected in April and August, respectively, from two regions of Mashhad and Fariman, Khorasan Razavi province, Iran. Then, they were transferred to the Herbarium of Faculty of Science, Ferdowsi University of Mashhad to be identified. According to differences in flowers and fruits and morphological differences, the botanist of the mentioned herbarium, identified *E. angustifolia* from Mashhad and Fariman as two different variants of the plant (Figure 1).

**Extraction**

Thirty grams of the fresh herb of each sample was extracted individually with 100 ml of ethanol and methanol for 3 h on a magnetic stirrer. The yielded solutions were filtered through Whatman no.1 paper and refrigerated at 4 °C for the future tests (kamkar et al., 2011 ▶).

**Determination of total phenolic content**

Total phenolic content of the extracts was determined using the Folin-Ciocalteau reagent (Horwits, 1984 ▶; Jahanban et al. 2009 ▶). Folin-Ciocalteau reagent was diluted 10 times with distilled water. The *E. angustifolia* extract solution (50 μL) was mixed with 1 ml diluted Folin-Ciocalteau reagent, 1 ml sodium bicarbonate solution (7.5%), and 2 ml distilled water. The mixture was incubated at room temperature for 15 min. The absorbance of the solution was determined at 730 nm using a spectrophotometer (Biowave, S2100, UK) and compared with gallic acid equivalents (GAE) calibration curve. The total phenolic content was expressed as mg gallic acid equivalents of 30 gram fresh *E. angustifolia* leaves and flowers**.**

**Determination of total flavonoid content**

Flavonoid content was determined according to the method described by Bonvehi et al. (2001) ▶ with some modifications. An appropriate dilution (50 μL) of the extract was mixed with 1 ml of 2% AlCl_3_ in methanol solution (5% acetic acid in methanol). The mixture was allowed to react for 10 min and the absorbance was read at 430 nm against a blank sample without reactants. Quercetine was used as the standard for the calibration curve. Total flavonoid content of the extracts was expressed as mg quercetin equivalents (QE) of 30 gram fresh *E. angustifolia*.

**Statistical analysis**

The results are expressed as mean ± SEM. Differences were analyzed using unpaired analysis of variance followed by t-test. For all analyses, p-value < 0.05 was considered statistically significant. Data were analyzed using Instat-N software.

## Results

**Figure 2:**
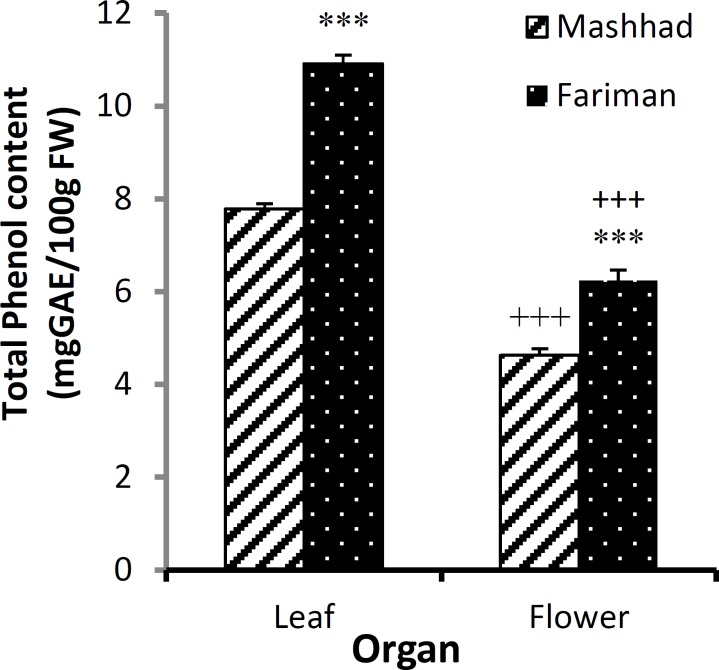
The amount of total phenolic content (mean ± SEM) in leaf and flower of *E. angustifolia *of Fariman and Mashhad variants using ethanolic solvent. Statistical significance for the difference between the data of Fariman* vs. *Mashhad variants: ***: p<0.001. Statistical difference between the data of flower *vs. *leaf (using unpaired t-test, n=4). +++: p<0.001

In this study, phenolic and flavonoids compounds from leaves and flowers in Fariman and Mashhad variants were measured using two solvents, ethanol and methanol (Tables 1 and 2). The phenolic compounds were more extracted by methanol (p<0.01 and p<0.001, Table 1) and flavonoids by ethanol (p<0.001, Table 2). The amount of phenolic compounds in the leaf of Fariman variant in both ethanolic (10.91±0.18 mgGAE/100gFW) and methanolic extract (10.28±0.36 mgGAE/100Gfw) was higher than Mashhad variant’s leaf (p<0.001, Figure 1 and p<0.01, Figure 2). Phenolic compounds of ethanolic extracts in Fariman variant’s flower were significantly higher than Mashhad variant’s (p<0.001, Figures 1). 

**Table 1:** Total phenolic content (mgGAE /100gFw) using ethanolic and methanolic extracts of two variety of *E. angustifolia* (for each group, n=4)

Solvent	Region	Leaf	Flower
Ethanol	Mashhad	7.78±0.11	4.63±0.14
	Fariman	10.91±0.18	6.24±0.22
Methanol	Mashhad	8.64±0.18‡‡	6.36±0.11‡‡‡
	Fariman	10.28±0.36	5.86±0.13

Statistical significance for the difference between the data of methanol *vs. *ethanol:

‡‡ : p<0.01,

‡‡‡ : p<0.001.

**Table 2:** Total flavonoid content (mgQE/100gFw) of two variety of *E. angustifolia* (for each group, n=4)

Solvent	Region	leaf	Flower
Ethanol	Mashhad	4.81±0.12	1.43±0.05
	Fariman	5.80±0.10	2.35±0.04
Methanol	Mashhad	3.34±0.0.05‡‡‡	1.34±0.05
	Fariman	3.36**±**0.0.05‡‡‡	1.34±0.06‡‡‡

Statistical significance for the difference between the data of methanol *vs. *ethanol:

‡‡‡ : p<0.001.

**Figure 3:**
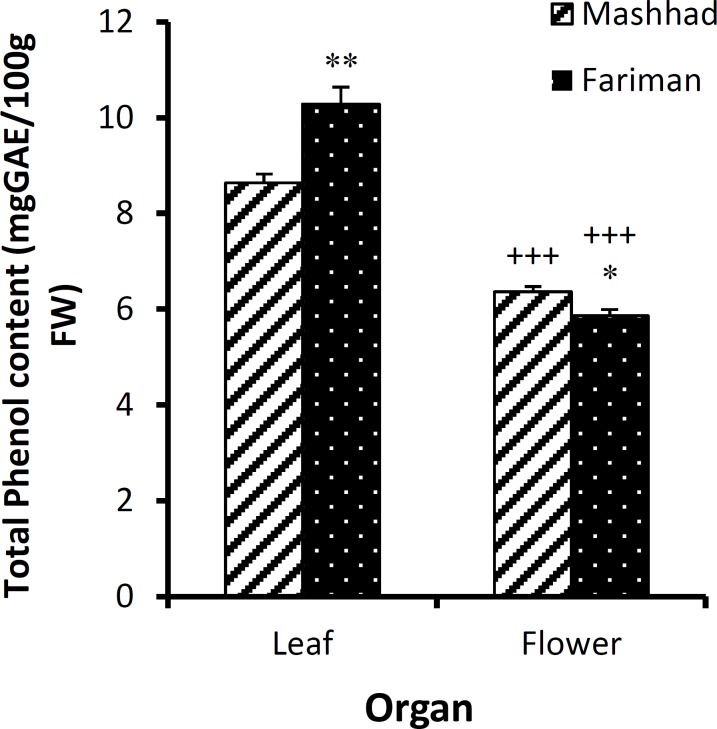
The amount of total phenolic content (mean±SEM) in leaf and flower of *E. angustifolia *of Fariman and Mashhad variants using methanol solvent. Statistical significance for the difference between the data of Fariman* vs. *Mashhad variants: *: p<0.05, **: p<0. 01. Statistical difference between the data of flower *vs. *leaf (using unpaired t-test, n=4): +++ : p<0.001.

**Figure 4:**
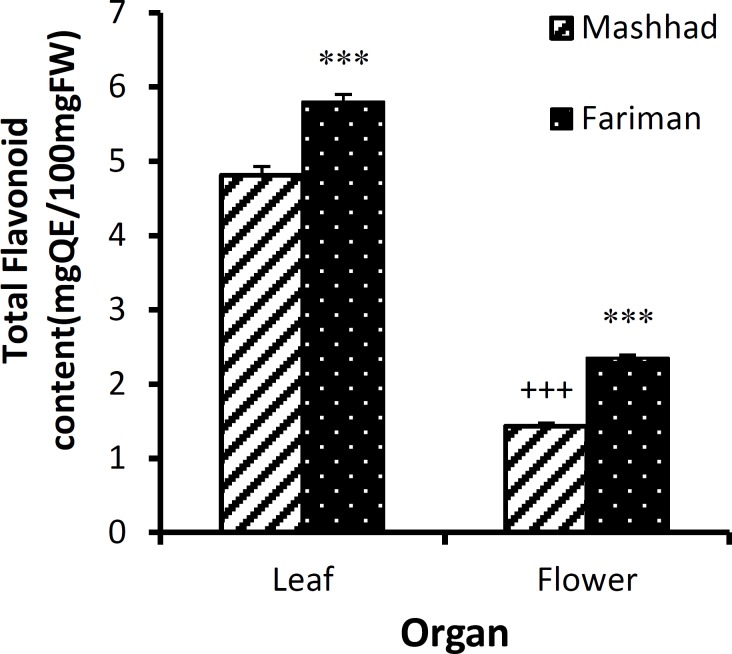
The amount of total flavonoid content (mean±SEM) in leaf and flower of *E. angustifolia *of Mashhad and Fariman variants using ethanol solvent. Statistical significance for the difference between the data of Fariman *vs. *Mashhad: ***: p<0.001. Statistical difference between the data of flower *vs. *leaf (using unpaired t-test, n=4): +++: p<0.001

**Figure 5:**
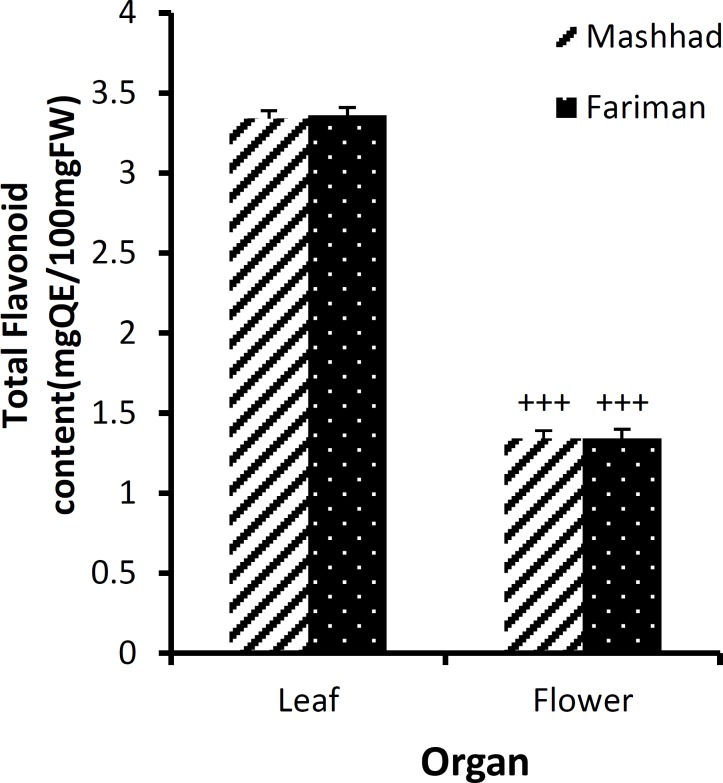
The amount of total flavonoid content (mean±SEM) in leaf and flower of *E. angustifolia *of Fariman and Mashhad variants using methanol solvent. Statistical difference between the data of flower *vs. *leaf (using unpaired t-test, n=4): +++: p<0.001

Flavonoid compounds of ethanolic extracts in leaves (5.80±0.10 mgQE/100gFw) and flowers (2.35±0.04 mgQE/100Gfw) of the Fariman variant showed significantly higher value than the ethanolic extract of leaves (4.81±0.12 mgQE/100gFw) and flowers (1.43±0.05 mgQE/100gFw) in Mashhad variant (p<0.001 for both case, Figure 3). However, no significant difference was observed in flavonoid content of methanolic extracts of the leaves and flowers between Fariman and Mashhad variants (Figure 4). In both variants and both solvents, phenolic compounds and flavonoids of leaves were higher than flowers (p<0.001 for all cases).

## Discussion

Phenols are very important plant constituents because of their radical scavenging ability resulting from their hydroxyl groups (Hatano et al., 1989 ▶). The phenolic compounds may contribute directly to the antioxidative action (Duh et al., 1999 ▶). It is suggested that polyphenolic compounds have inhibitory effects on mutagenesis and carcinogenesis in humans (Tanaka et al., 1998 ▶). Flavonoids and flavones are widely distributed secondary metabolites with antioxidant and antiradical properties (Makari et al., 2008 ▶). Alexandru et al. determined the flavonoid and total phenolic content of several herbs including hyssop and yarrow flowers (Alexandru et al., 2007 ▶). 

The antioxidant activity of natural sources is due to the active compounds present in the plants. The most natural phenol and flavonoid contents can be found in leaves, fruit, flower, and seeds of plants. Most of these compounds are natural phenols or polyphenols, e.g., tocopherols, flavonoids, and derivatives of cinnamic acid, phosphatidic, and other organic acids. It is well known that the antioxidant activity of plant extracts containing polyphenol components is due to their capacity to donate hydrogen atoms or electrons and scavenge free radicals (Sreedam et al., 2010 ▶). 

In this study, the total phenol and flavonoid content of two *E.* *angustifolia* variants were compared. Results showed that the amount of phenolic compounds and flavonoids in both ethanolic and methanolic extracts were higher in Fariman variant than Mashhad variant. Many factors including climate, soil and height are involved in levels of plant secondary metabolites such as phenolic compounds. This difference might be associated with ecological conditions (Schwazt et al., 2009 ▶). In fact variations in developmental responses of *E*. *angustifolia *leaves to spatial heterogeneity was shown which could be related to its ecological strategies (Klich, 2000 ▶). 

In this study, two solvents, ethanol and methanol, were used in order to extract phenolic and flavonoids compounds from leaves and flowers of Fariman and Mashhad variants. We observed that phenolic compounds were extracted better by methanol and flavonoids with ethanol solvent. Different solvent extractions provide different types of compounds because of their variable chemical nature and sensitivity toward extraction or hydrolysis methods (Jahanban Isfahlan et al., 2010 ▶). Among different solvents commonly used for extraction of plant material for polar and non-polar compounds, methanol and ethanol are the most effective ones. Therefore, any measurement with these alcoholic extracts can extract polar and non-polar compounds and thus leads to more antioxidant properties (Harborne, 1998 ▶). In addition, ethanol is reported to be a better solvent to extract phenolic compounds from *persimmon* compared with other solvents (In-Cheol et al., 2010 ▶). 

Bang and colleagues (1999) ▶ showed that using methanol for extraction shows more total phenolic content compared with aqueous solvents and even the concentration of the used methanol can 

affect the extraction of plant content. In other words, increasing the concentration of methanol increases the amount of extracted phenol. Ethanolic and methanolic solvents were used in several studies previously, and the results showed a different outcome of the two solvents (Mamashloo et al., 2012 ▶,) which support the results of the present study. Most phenolic compounds and flavonoids were found in the leaf extract. Due to photosynthesis in leaves, flavonoid biosynthetic pathway precursors (Shikimic acid) are more abundant in leaves than in other organs. This is also another reason for the higher amount of flavonoids in leaves compared with flowers and other organs (Andersen and Markham, 2006 ▶). 

Dokhani and colleagues (2005) ▶ also reported the total amount of phenolic compounds in *A. eriophora* to be higher in leaf extract than flowers. Lowest flavonoid compound was observed in methanolic flower extract of both variants. Ayaz et al. in 1999 ▶ reported the presence of 4-hydroxybenzoic acid, phenolic acid, and acid caffeic in *E. angustifolia *fruit (Ayaz et al., 1999 ▶). In another study significant amounts of flavonoid, terpenoid, and cytosterol compounds were derived from *E. angustifolia *leaves (Dembinska-Migas and Gill, 1973 ▶). 

The content in polyphenolic compounds (flavones and polyphenolcarboxylic acids) in the soft extract of *Elaeagnus angustifolia *L. (oleaster) young branches, using high-performance liquid chromatography coupled with mass spectrometry (HPLC-MS) were also shown in two different types of extract (Bucur et al, 2009 ▶). Results of the present study are in accordance with previous studies in regard to the presence of phenolic and flavonoid compounds. Many synthetic antioxidant compounds have toxic effects or cause cancer which have turned attentions to the natural antioxidants and phenolic compounds (as a member of a family of natural antioxidants) that participate directly in anti-oxidative activities (Duh et al., 1999 ▶). Evidence suggests that increased intake of dietary antioxidants, fruits, or vegetables with anti-oxidative properties improves the quality of life, delays the onset, or reduces the risk of degenerative diseases associated with aging (Siriwardhana and Shahidi, 2002 ▶).

The results of the present study showed that in both varieties and solvents, the amount of phenolic and flavonoids compounds in leaves were higher than flowers. In addition, the phenolic and flavonoids compounds were higher in Fariman compared to Mashhad variants.

## Conflict of Interest

There is not any conflict of interest in this study.
